# Special Issue “Molecular Links Between Sensory Nerves, Inflammation, and Pain 3.0”

**DOI:** 10.3390/ijms27198521

**Published:** 2026-09-24

**Authors:** Éva Szőke, Zsuzsanna Helyes

**Affiliations:** 1 Department of Pharmacology and Pharmacotherapy, Medical School & Centre for Neuroscience, University of Pécs, 12 Szigeti Street, H-7624 Pécs, Hungary; 2 Hungarian Research Network, Chronic Pain Research Group, University of Pécs, 12 Szigeti Street, H-7624 Pécs, Hungary; 3 National Laboratory for Drug Research and Development, Magyar Tudósok Krt. 2, H-1117 Budapest, Hungary; 4 PharmInVivo Ltd., Szondi Str. 10, H-7629 Pécs, Hungary

Pain is a complex sensation that emerges from dynamic interactions of sensory neurons, immune and inflammatory mechanisms, membrane signaling processes, and central nervous system networks. Although acute nociception is essential for protection from tissue damage, persistent activation or maladaptive plasticity within these systems may lead to chronic pain, which remains a major clinical challenge. The primary nociceptive neurons have a significant role in pain perception, and their capsaicin-sensitive peptidergic sensory nerve endings have triple functions. The first, afferent function is the transmission of sensory and pain signals to the central nervous system. Pro- and anti-inflammatory neuropeptides and other mediators like purines are also released from the activated nerve endings, which exert local efferent functions on vessels and immune cells and systemic efferent functions reaching the circulation. These nerves are involved in several pathophysiological conditions and diseases without appropriate therapy. The complex sensory-vascular-immune interactions have significant regulatory roles in a range of inflammatory and pain conditions. Increasing evidence indicates that pain cannot be understood solely as a consequence of neuronal excitation: inflammatory mediators, immune cells, metabolic alterations, membrane microdomains, and central modulatory circuits all contribute to the initiation, amplification, and persistence of painful states. The Special Issue “Molecular Links between Sensory Nerves, Inflammation, and Pain 3.0” brings together studies that illustrate this multidimensional nature of pain research. The contributions range from developmental analgesia and membrane lipid regulation of nociceptive ion channels to inflammatory biomarkers in diabetic neuropathy, experimental models of provoked vulvodynia, and central modulation of pain associated with dopaminergic neurodegeneration. These papers highlight how studies at molecular, cellular, systemic, and behavioral levels may identify new opportunities for safer and more targeted pain therapies.

An important clinical challenge is pain management during early postnatal life. Logashkin and colleagues investigated dexmedetomidine as a short-term analgesic intervention in the immature nervous system [1]. Effective analgesia in neonates, including premature infants, is particularly difficult because pharmacological suppression of pain must be balanced against the vulnerability of the developing brain. Dexmedetomidine is already used as an adjunct to anesthetic regimens in neonatal and pediatric practice, but an important unresolved question is whether its actions interfere with neuronal activity patterns that are critical for normal brain maturation. Using neonatal rats, the authors examined its pharmacodynamic effects on neuronal as well as cardiorespiratory functions. Dexmedetomidine produced pronounced analgesia while only weakly modifying immature cortical and hippocampal network activity and sleep-cycle physiology. Although modest reductions in respiratory and heart rates were observed, the findings support further consideration of dexmedetomidine as a potentially useful short-term strategy for analgesia in the immature nervous system. This work emphasizes that the evaluation of analgesic efficacy in early life should extend beyond pain suppression itself and include the functional integrity of developing neuronal networks.

At the peripheral sensory level, Horváth and colleagues explored a fundamentally different mechanism: the regulation of nociceptive ion channels by the lipid environment of the plasma membrane [2]. Their study focused on the thermo- and pain-sensitive Transient Receptor Potential Melastatin 3 (TRPM3) and TRP Melastatin 8 (TRPM8) channels and the functional importance of lipid rafts. Previous observations had indicated that depletion of cholesterol or sphingomyelin, or inhibition of sphingolipid biosynthesis, can reduce native TRPM8 channel opening in cultured sensory neurons while leaving TRPM3 relatively unaffected. The authors therefore examined lipid raft disruption both in vitro and in acute nocifensive mouse models. Sphingomyelinase and methyl-beta-cyclodextrin reduced icilin-induced nocifensive behavior, although the same interventions did not directly inhibit recombinant TRPM3 or TRPM8 channels expressed in heterologous cells. These findings suggest that the effects are dependent on the native membrane environment rather than reflecting simple direct channel antagonism. The study thus reinforces the concept that membrane cholesterol and sphingolipids are active regulators of sensory transduction. Importantly, sphingolipid cleavage appeared more effective than cholesterol depletion, pointing toward membrane microdomain remodeling as an unconventional but potentially exploitable approach to peripheral analgesia.

The close relationship between inflammation, metabolic disease, and neuropathic pain is addressed by Stoian and colleagues in their review of biomarkers associated with chronic inflammatory processes underlying diabetic neuropathy [3]. The increasing prevalence of type 2 diabetes mellitus makes peripheral diabetic neuropathy an especially important cause of chronic pain and disability. The review highlights the convergence of insulin resistance, oxidative stress, endothelial dysfunction, and persistent low-grade inflammation in the development and progression of diabetic complications. Classical inflammatory mediators such as C-reactive protein, interleukins, and tumor necrosis factor-α are discussed alongside NF-κB, Toll-like receptors, caveolin-1, and monocyte chemoattractant protein-1. These pathways provide a molecular framework linking metabolic disturbance to inflammatory gene expression, vascular dysfunction, and peripheral nerve damage. The authors also draw attention to macrophage involvement and to the association between vitamin D deficiency and peripheral diabetic neuropathy. The reported potential of vitamin D supplementation to reduce neuropathy severity and inflammatory markers illustrates how biomarker research may eventually inform therapeutic stratification. At the same time, the review appropriately emphasizes the need for larger clinical studies investigating anti-inflammatory agents, TNF-α-directed approaches, antioxidants, and other interventions capable of modifying the inflammatory component of painful diabetic neuropathy.

Another chronic pain condition in which inflammatory and neuroimmune mechanisms are likely to be crucial is localized provoked vulvodynia. Nakhleh-Francis and colleagues provide a systematic overview of animal models used to investigate this complex disorder [4]. Human studies of vulvodynia are inherently challenging because of its multifactorial etiology and the difficulty of dissecting individual pathogenic mechanisms in patients. Experimental models, therefore, provide an essential platform for identifying causal pathways and testing therapeutic concepts. The authors identify three principal categories of animal models—inflammation-induced, allergy-induced, and hormone-induced vulvodynia—and describe several agents capable of producing vulvodynia-like phenotypes through distinct mechanisms. Particularly relevant to the theme of this Special Issue are observations involving hyperinnervation, mast-cell proliferation, other immune-cell populations, inflammatory cytokines, and neurotransmitter signaling. These models demonstrate how altered sensory innervation and local immune responses can interact to generate persistent hypersensitivity. By organizing the available experimental approaches, this review provides a valuable framework for selecting appropriate models and for interpreting apparently heterogeneous findings across studies. A better mechanistic understanding of these models may ultimately facilitate the development of preventive strategies and more specific treatments for a condition for which effective therapeutic options remain limited.

Zhang and colleagues extend the scope of the Special Issue from peripheral and inflammatory mechanisms to central pain modulation in Parkinson’s disease [5]. Loss of midbrain dopaminergic neurons is a defining pathological feature of Parkinson’s disease, and chronic pain affects a substantial proportion of patients. The pedunculopontine tegmental nucleus (PPTg), located in the pontomesencephalic tegmentum, participates in motor control and sensory integration and has also emerged as a potential target for deep brain stimulation. Using mice with 6-hydroxydopamine-induced lesions of midbrain dopaminergic neurons, the authors investigated whether dopaminergic damage produces pain hypersensitivity and whether chemogenetic activation of PPTg neurons can modify this phenotype. The lesion induced pain hypersensitivity with only a minor impairment of voluntary movement and without detectable disruption of sensorimotor function in the adhesive tape removal test. Importantly, hypersensitivity was alleviated both by levodopa and by chemogenetic activation of the PPTg. These results identify the PPTg as a component of the circuitry capable of modulating pain associated with dopaminergic neurodegeneration and suggest that central neuromodulatory strategies may complement conventional pharmacological approaches in Parkinson’s disease-related pain.

Taken together, these papers illustrate the remarkable biological diversity of mechanisms that converge on pain. They also reveal several recurring principles. First, the activity of sensory neurons is strongly shaped by their molecular and cellular environment. This is evident in the modulation of TRPM8 signaling by membrane cholesterol and sphingomyelin and in the interactions among inflammatory mediators, immune cells, and peripheral nerves in diabetic neuropathy and vulvodynia. Second, effective pain control requires attention to physiological context. The developing nervous system may respond differently to analgesic interventions than the adult nervous system, making simultaneous assessment of analgesia, neuronal network function, and cardiorespiratory effects essential. Third, pain is not exclusively a peripheral sensory phenomenon. The study of the PPTg in a model of dopaminergic neurodegeneration illustrates how central circuits involved in motor and sensory integration can profoundly influence pain behavior. Finally, these studies collectively demonstrate the value of combining molecular and cellular experiments with disease-relevant animal models and clinically oriented observations. The *International Journal of Molecular Sciences* team and the two guest editors hope that the studies and reviews collected here will stimulate further interdisciplinary research into these molecular links and contribute to the development of mechanism-based strategies for the prevention and treatment of pain.
